# Understanding the Experience and Perspectives of Parkinson's Disease Patients' Caregivers

**DOI:** 10.1155/2019/3082325

**Published:** 2019-01-31

**Authors:** Tamene Keneni Walga

**Affiliations:** Department of Psychology, College of Social Sciences & Humanities, Debre Berhan University, Debre Berhan, Ethiopia

## Abstract

This research sets out to explore, uncover, and understand the experiences and perspectives of people who care for patients with Parkinson's disease (PD). To this end, 20 participants who accompanied patients with PD to a training organized by Parkinson Patients Support Organization-Ethiopia (PPSO-E) provided the data required. Analysis of the data produced several themes such as delay in PD diagnosis and intervention, differing reactions to PD diagnosis, toughness of caring for PD patients, community's limited understanding and distortion of PD, lack of specific name and clear expression for PD in local languages, lack of sufficient support to the caregivers, caregivers' compassion and patient's courageousness, and shortage and expensiveness of PD prescriptions. The themes produced have been discussed in light of existing literature. Based on the findings of this research, recommendations were forwarded and direction for future research was indicated.

## 1. Introduction

Parkinson's disease (PD), named as such after James Parkinson who described the disease for the first time in 1817, is defined in many ways. WHO [1] defines Parkinson's disease (PD) as “a chronic progressive neurodegenerative disorder of insidious onset, characterized by the presence of predominantly motor symptomatology (bradykinesia, rest tremor, rigidity, and postural disturbances)” (p. 140). It is a chronic irrepressible neurological disorder afflicting mainly adults above 50 years of age [2]. “PD is a complex neurodegenerative progressive disease process that results in a broad spectrum of clinical manifestations such as rigidity and tremors that affect mobility…” [3, p. 1].

PD is one of the least known diseases in Africa in general and in Ethiopia in particular. This does not mean that it is not prevalent in Africa and in Ethiopia. It might be due to scarcity of research and lack of appropriate diagnostic criteria. Since it is one of the least studied illnesses, its rate of prevalence and incidence in Ethiopia is not well documented. There are few exceptions, however. For example, an earlier door-to-door prevalence study documented seven cases in 100,000 people [4]. In a relatively recent review of referral hospital admissions, only about 7% ( 46 out of 720 referrals) of those referred to a neurological clinic for having motor disorder have been diagnosed to have PD [5].

PD poses multifaceted challenges not only to the patients themselves but also to those around them including caregivers. In fact, Kibra [6] describes PD as a challenging journey that involves the patient, her/his caregivers, family members, and close relatives and friends among others. Helen [7] has found out that PD patients in Ethiopia and their family members and close relatives and friends face multilayered economic and psychosocial problems such as early retirement, self-stigmatization, lonesomeness, and social anxiety. Abenet, Mellon, and Fikre [8] have shown that both motor and nonmotor symptoms of PD affect health-related quality of life among PD patients with the effect worsening with the increasing severity of the disease. Gultekin, Ekinci, Erturk, and Mirza [9] have concluded that caring for PD patients compromises caregivers' psychological well-being. Furthermore, based on her/her scoping review, Bhimani [3] concluded that PD patients' caregivers experience a wide range of caregiving-related stresses that need to be recognized and understood. Ferreira, Coriolano, and Lins [10], in their integrated review, have come to conclude that “the challenge of caring for a person with PD needs to be recognized by caregivers, professionals and health managers as a tool for the (re)construction of care” (p.1). Overall, caregivers are matchless assets not only for the PD patients but also for healthcare providers and thus their concerns, experiences, and perspectives are of greater importance and relevance. However, the study of PD patients' caregiving and its challenges from caregivers' perspectives is scarce globally and it is totally lacking in Ethiopia to the researcher's best knowledge. In effect, study of the PD itself and its patients is scarce. With the aim to help fill the gap and paucity in research into the issue, the main purposes of this research were to explore, uncover, and understand the lived experiences and perspectives of selected PD patients' caregivers.

## 2. Methods and Materials

### 2.1. Research Questions

In order to achieve the purposes put forth, this research sets out to answer the following research questions:

1. What does it mean to be PD patient's caregiver?
2. How is PD perceived and understood by the society? What about its cause?
3. What is PD called by the society?
4. To what extent are the caregivers under study felt supported?
5. How do the caregivers cope with burdens of caring for PD patient under their care?

### 2.2. Research Design

A mixed research with predominantly qualitative aspect was used to explore, uncover, and understand participants' personal and subjective experiences and perspectives of caring for PD patients. More specifically, a descriptive phenomenological qualitative research was used to capture the richness and complexity of being a caregiver to PD patients.

### 2.3. Participants

Data for this research came from caregivers of PD patients. Convenience sampling technique was used to select the participants of this study in that almost all the caregivers who accompanied the PD patients who came to attend a training organized by Parkinson Patients Support Organization-Ethiopia (PPSO-E) provided the data required. PPSO-E is, to the researcher's best knowledge, the only nongovernmental organization (NGO) working with patients of PD in Ethiopia. Initially, it was established as a self-help association and later changed into an indigenous NGO. PPSO-E envisages seeing patients of PD in Ethiopia and their caregivers lead better and meaningful life and its mission is to look for ways that inform patients of PD and their caregivers through the provision of information and economic and psychological support [6].

### 2.4. Data Collection Instrument and Procedure

A mostly qualitative survey questionnaire was used to collect the data. The questionnaire consisted of four parts. The first part of the questionnaire consisted of close-ended questions that were used to capture caregivers' and patients' demographic information. The second part contained open-ended questions related to PD, PD patients, and their care. Questions in the third part were Likert-type questions that asked the participants to evaluate (rate) supports available to them in caring for the patient under their care. The questions were answered on a ten-point scale ranging from 1 (very low) to 10 (very high). The fourth part had two open-ended questions that asked the participants to add any information or idea deemed relevant to them.

The questionnaire was administered to participants with the help of PPSO-E staff in a waiting room where caregivers stayed when the patients they accompany were attending training. The questionnaire was administered to five of the participants who cannot read and write in a form of interview by the researcher. The researcher tried his best to elicit as much information as possible from these participants. Participation in this research was completely voluntary and oral consent was obtained from each participant. The purpose of the research was explained to the participants both in the questionnaire and orally.

The questionnaire was prepared and administered in Amharic to allow the participants' maximum understanding of the questions and provide richer and accurate data. Participants were given enough time and asked and encouraged orally to provide as much information they were comfortable to give.

### 2.5. Data Analysis

Data analysis in qualitative research in general and descriptive phenomenological research in particular is a cyclical process in that the researcher is required to move back and forth between steps as suggested by Creswell [11] and Patton (2002). To this end, following Walga [12], analysis of the nonnumerical data in research was conducted as follows. First, participants' responses to the open-ended questions were read several times and a separate sheet of paper was used to make notes of anything that appeared to be significant and of interest. The second stage involved returning to the participants' responses anew and using the notes taken on the separate paper to transform the responses and notes into more specific themes, which were expressed in a form of phrase or short statement. The third stage involved further reduction of the textual data, which were then given descriptive label that conveys the conceptual nature of the themes therein. Finally, the themes were described textually and then supported and substantiated by appropriate quotes and excerpts from participants' responses. For the numerical data, descriptive statistics such as percentage and mean were computed and shown in appropriate graphs and figures.

## 3. Results

### 3.1. Characteristics of the Participants

Table 1 summarizes the demographic characteristics of the caregivers studied. The participants were given codes instead of their real identity for the sake of confidentiality.

As can be seen from the table, ages of the caregivers ranged between 14 and 66 years showing that a 14-year-old school boy and a 66-year-old adult who were supposed to be cared for were shouldered with the responsibility of caring for a PD patient. In fact, about 40% of the caregivers were young persons below the age of 25 years. In terms of gender, caregivers were disproportionately females for they accounted for 65% of the participants. This shows that women shoulder the responsibility of caring for PD patients more than men do. Although this sample is a convenience sample and is very small in size, it is very likely that caregiving for patients with chronic conditions including patients with PD is feminized in Ethiopia. Caregivers were also people from different walk of life including school and university students. About 50% of them were not engaged in any income-generating activity. In terms of education, people with different level of education involved in caring for PD patients included people who cannot read and write and people who hold university degree. In terms of civil status, majority of the caregivers (about 60%) were single young people.

**Figure 1:**
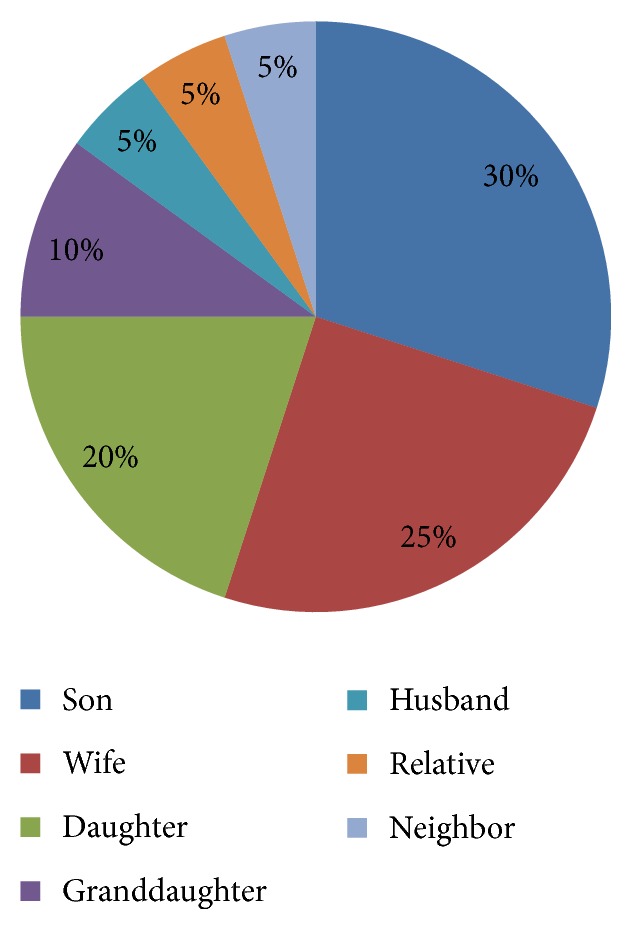
Caregivers' relationship with the PD patients.

Figure 1 shows that nearly a third of PD patients were cared for by their sons followed by wives and daughters at 25% and 20%, respectively. Granddaughters constituted about 10% of the PD caregivers. The rest of PD patients were cared for by husband (5%), relative (5%), and neighbor (5%).

**Figure 2:**
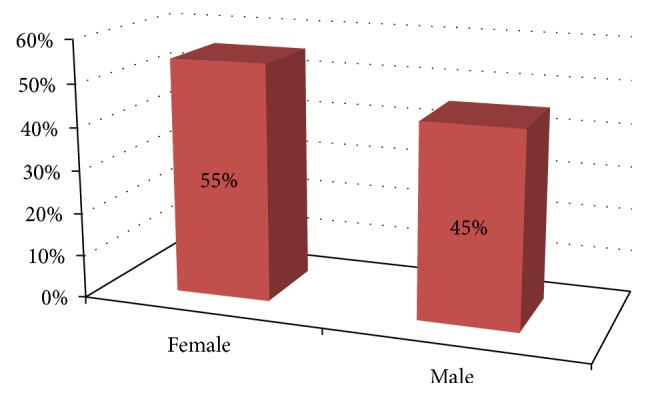
PD patients by gender.

Figure 2 shows the PD patients under the care of the participants of this research by gender. As can be observed from the figure, women accounted for 55% of them. The age of PD patients ranged between 50 and 89 (mean = 66.72; SD = 7.91). Vast majority (75%) of them were married. Almost all of the caregivers do not know the exact time when the illness of the PD patient under their care started the but in average it was about 5 years since the patients were diagnosed with PD.

### 3.2. Delay in Diagnosis and Intervention

Delay in diagnosis and intervention was a recurrent theme in the data collected. This is clearly illustrated by the excerpts taken from the responses given by some participants regarding the diagnosis process they went through.

Her illness was not known for so long time… (*P*18).

This disease hurts slowly. It started her many years ago but we could learn it is PD very recently (*P*09).

For many years, he used to work with his illness. But, later he stopped working because he couldn't. Two years ago, we went to Tikur Anbessa Hospital and they told us his disease is PD (*P*14).

First, she was involved in a car accident and hurt her back. She then showed strange symptoms as result of which we visited several hospitals. After many examinations, we were told that she developed a PD (*P*16).

After visiting hospitals for so long time, we were told that he's a PD patient (*P*12).

Some caregivers also reported not only a delay in diagnosis but also a sort of misdiagnosis as the three quotes below demonstrate:

Since he had other illnesses including diabetes and hypertension, the symptoms he showed were taken for granted to be that of his earlier illnesses. As a result of this, we could know that he had a PD after many years. We visited several hospitals before we could learn he's a PD patient (*P*13).

It was not known at once. She made so many examinations. At first, her disease was misdiagnosed to be another illness. We visited so many hospitals. Lastly, we were told it is a PD (*P*03).

She's diabetic; we were misled and confused by this and she underwent examination too late (*P*06).

### 3.3. Differing Reactions to PD Diagnosis

Caregivers' reactions to the results of PD diagnosis of the PD patient under their care differed mainly along three lines. While majority of the caregivers reported that they were terrified, so sad, so sorrowful, or disappointed upon learning that their beloved one had a PD, others were at ease because they had no knowledge and information about PD. Yet, few caregivers tried to embrace it.

As the quotes that follow exemplify, majority of the participating caregivers reacted negatively to incident:

What I felt then was, since she is my mother, I was so sad; that sadness is still fresh in my heart and mind (*P*20).

We were very sorrowful (*P*02).

We felt deep inside; I was terrified by the news; the whole family got terrified, too. I asked myself why this disease to us!(*P*01).

We were distressed; we felt so tense; we cried; since we learned that it is an incurable disease, we felt so sorrow!(*P*16).

We were distressed when we learned that the disease is not curable (*P*12).

We were disappointed so much!(*P*08).

On part of me, upon learning that he has had a PD, I couldn't believe it because this disease comes from a curse….(*P*03).

It's difficult; we couldn't believe it; we were so desperate upon learning that the disease is not curable (*P*09).

Some participants stated that they were at ease upon learning that their beloved one had a PD because they had no knowledge and information about PD. This is typified in the excerpts taken from questionnaire responses below:

I was too young at that time. I thought the disease is curable by some sort of medication. We had no understanding about PD. Perhaps, that is why were at ease at that time (*P*19).

At that time we had no knowledge of the disease that we have today. So we were not distressed; we were at ease a little bit (*P*11).

We had no knowledge and understanding of the disease at that time; we had no unusual feeling upon learning that he had a PD (*P*13).

Yet, as the excerpts that follow epitomize, few caregivers tried to embrace the problem and convinced themselves to provide any care in their ambit:

Although we knew that the disease is difficult, we accepted it; that was what you can do as a caregiver (*P*07).

….when you know that the disease is PD and when you learn that it is incurable, you convince yourself and strive to help the patient as much as you can…(*P*17).

### 3.4. Caring for a PD Patient is a Tough/Difficult Undertaking That Requires Patience

One of the key questions that have been posed to the caregivers was the following: what does it mean to be a PD patient's caregiver? They were asked to describe it with a single word or phrase. Almost all of the participants (caregivers) admitted being a caregiver to a PD patient is a difficult undertaking in one way or the other. The following excerpts from the participating caregivers' responses show either explicitly or implicitly how much caring for a PD patient is difficult and challenging. In addition, the excerpts show how much patience is required on part of the caregiver.

Being forbearing pays off! It requires being too much tolerant!(*P*16). It means being too much forbearing (*P*16). It means caring for the patient with greater tolerance (*P*19).You need to be very much forbearing and accepting (*P*17).It demands too much patience (*P*04).

It means understanding the patient's feeling and helping him accordingly! Is that easy? No! It is not easy!(*P*15).

It demands you to be very persevering because you care for a person who is healthy and sickat the same time (*P*11).

It's tough! Though difficult, it doesn't bother me because he's my children's father (*P*14).

To be frank, it is very difficult! But, is she not a mother! You bear it (*P*02).

It's very difficult! Those who experienced it know how much it's difficult (*P*03).

It's something that challenges your patience (*P*08).

It's difficult (*P*09).

It's very difficult. No one is beside me to help me. On top of that he always complains!(*P*10).

Although it is difficult, you bear it as far as he is living (*P*12).

It's difficult! But, since the patient bears all the pains you need to bear it (*P*13).

### 3.5. Caring for PD Patient is a Shared Responsibility

Interestingly, responses from the participants to the question “are you the only caregiver to the patients?” show that overwhelming majority of the caregivers (about 75%) had at least another person who helps them with the caregiving. For instance, P19 who cares for her father said her brother helps her a lot. P12 who cares for her husband said that her children share their caring responsibility during their spare time. Another participant (P14) who cares for husband also admitted that her children help her with the caregiving undertakings during their spare time.

### 3.6. Community's Limited Understanding and Distortion of PD

In the participants' opinion, their community's understanding of PD is very low and even distorted. This theme is clearly reflected in the quotes hereunder. What have been stated by P05, P03, P16, and P19 were clear indicators of how much PD is distorted.

The community sees PD as a punishment from God; it is also seen as a consequence of one's sin; even many people believe that PD is caused by hitting (killing) a cat with one's hands as child (*P*05).

Our community's understanding of PD is very low; they don't understand it even when we tell them (*P*07).

This disease is not widely known in our community….(*P*09).

As regards PD, majority of our community does not have enough understanding of it; some individuals may have limited information from media such as television; still majority lacks understanding; even there are people who believe that PD comes from a curse or evil spirit (*P*03).

They don't have enough understanding of it; for that matter, I can say they don't have at all for they believe that PD is inherited from ancestors; some even go to the extent to say that the patient killed a cat as a childand contracted the disease (*P*19).

They see it as a curable disease like other illnesses… (*P*14).

Majority does not know about PD…; your mother's hands quiver; did she kill a cat as a child? People ask me this question often (*P*16).

### 3.7. Lack of Specific Name and Clear Expression for PD in Local Languages

How is PD called in your community? Participants' responses to this question showed that PD has no specific name and clear expression in local languages that the participants speak. About 60% of the participants said that the disease is called and is known as “*nervbeshita*” or simply “*nerv*” in their locality, which can be translated as* disease of nerve *and* nerve*, respectively. Although the first expression is closer to the definition of PD in literature, it does not convey clear message. Some participants said it has no name. Others said they do not know what it is called locally. Overall, based on the responses provided by the participants, PD has no specific and clear name in local languages.

### 3.8. Lack of Sufficient Support to the Caregivers

**Figure 3:**
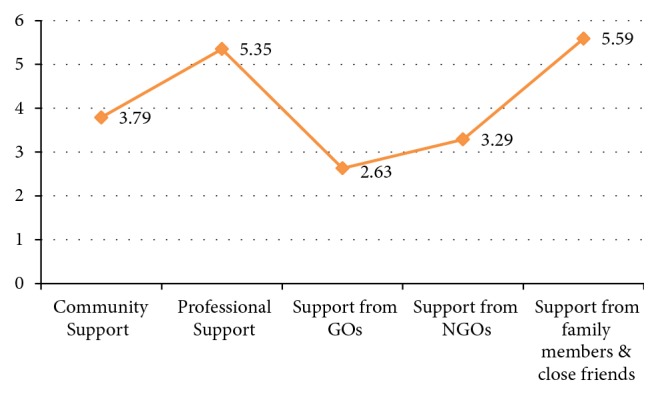
Level of support from each source of support.

Participants of this study were asked to rate supports they were supposed to get from the larger community, concerned professionals, governmental organizations (GOs), nongovernmental organizations (NGOs) and faith-based support groups, and family members and close friends on a ten-point rating scale ranging from 1 (very low) to 10 ( very high). Average scores for all sources of support were shown in Figure 3.

As can be seen from the figure (Figure 3), overall, the caregivers under study were not getting adequate support from all sources. However, as reported by the caregivers, they were getting generous support from family members and close friends and professionals (e.g., individual neurologists and general practitioners) at 5.59 and 5.35 out of possible 10 points, respectively. Supports from other sources such as the larger community, nongovernmental organizations, and governmental organizations (e.g., health department at all levels, departments of labor and social affairs) were below the midpoint with support from governmental organizations being the least at 2.63 out of 10. As far as support from NGOs is concerned, participants pointed out that PPSO-E is the only NGO that stands with them and they acknowledged and appreciated PPSO-E's rare support.

In addition to rating the supports, few participants elaborated on the supports they get from different sources. For instance, P01 appreciated the support from professionals as “…*they support us; they do what they can do for us; it is good*.” The same participant also cherished the support that the PD patient under his care gets from his close relatives and friends as “…*their support is very fine; all his friends come together and visit him; and they encourage him, too; so it's good*”(P01). On the other hand, a 60-year-old participant who has been caring for her husband for more than 12 years lamented the lack of support from the community by asking the question: “*Do people who live in the same neighborhood help each other these days*?” This same participant complained from the lack of support from public agencies such as health facilities by saying “…* for example, when we go to health stations around to look for PD prescriptions, they always say they don't have and direct us to private drug stores where the prescriptions are very expensive…; that is how they help us*” (P10). P10 also seems unhappy in the support that PD patients get from close others when she says “…*only two of his former friends visit him occasionally and give him some coin to help us buy prescriptions*.”

### 3.9. Caregivers' Compassion and Patient's Courageousness

It is unquestionable that caring for PD patients is a burden and stress that requires some sort of coping strategies and/or resources. I posed the question “how do you cope with the burden/stress of caring for the PD patient?” to the participants. Their responses to this question mainly reflected two key resources that helped them to cope with the burden of caregiving as evident in the excerpts hereunder. These two key resources were caregivers' compassion and patient's courageousness.

I see caring for him as God-given duty and responsibility; he didn't imagine that he'd acquire this disease. He is a very clever person. I'll be by him as far as I can forever (*P*03).

Thanks to God! I bear it; he also is of unimaginable stamina; he doesn't give up at all (*P*13).

He is very courageous! He is always bold and doesn't give up-that encourages and strengthens me (*P*14).

I am happy to be able to help her; helping her doesn't bother me for I put myself in place of her (*P*06).

Embracing the patient and his/her behaviors helps a lot (*P*11).

### 3.10. Shortage and Expensiveness of PD Drugs

Finally, participants of this study added that shortage and expensiveness of PD drugs are what make their caregiving responsibility very challenging as typified in the responses below. Going from one drug store to another to look for PD drugs was what the participants lamented unanimously.

When we go to health stations around to look for PD prescriptions, they always say they don't have and direct us to private drug stores where the prescriptions very expensive (*P*10).

The number of PD patients is on rise and so is the demand for PD drugs… I wish enough supply of PD drugs (*P*19).

PD has no curing drugs though there are drugs that kill its symptoms. However, these drugs are not available with reasonable price (*P*01).

What I want to add is there is short age of PD drugs supply; in addition, the drugs available are very expensive. We need urgent support in this regard (*P*16).

## 4. Discussion

The main purposes of this study were to explore, uncover, and understand the experiences and perspectives of the caregivers of persons with PD. To this end, 20 primary caregivers provided the data required. Careful analysis of the responses obtained yielded the following key themes:

- Delay in diagnosis and intervention
- Differing reactions to PD diagnosis
- Caring for a PD patient is a tough undertaking that demands patience
- Caring for PD patients is a shared responsibility
- Community's limited understanding and distortion of PD
- Lack of sufficient support to the caregivers
- Caregivers' compassion and patient's courageousness
- Shortage and expensiveness of PD drugs/prescriptions

Delay in diagnosis and intervention seems to emanate mostly from shortage of information and knowledge on PD among the PD patients, their caregivers, and the larger public. In fact, Kibra [6] has reiterated the grave lack of information on PD and how this lack of information has negatively affected the lives of PD patients and their caregivers. Early identification and intervention of PD is very crucial for it may slow down the progression of the disease and its symptoms. Although curing PD is not yet possible, the treatment of its symptoms has been improving over time. Levodopa and dopamine agonists are some of the common pharmacotherapies used to treat PD in Ethiopia and around the globe [13]. Rehabilitation and physical exercises are often recommended to slow down the motor symptoms of PD [14]. The efficaciousness of these pharmaco- and physical treatments is contingent upon early diagnosis, identification, and intervention. Early diagnosis is likely to lead to early identification and intervention. The earlier the intervention is, the more efficacious the symptomatic treatments of PD are. In line with this assertion, Dubayova et al. [14] suggested that early help-seeking behavior in PD patients may result in a greater chance of limiting impairment of their quality of life.

Differing reactions to PD diagnosis were another theme that emerged from the current data. Majority of the caregivers in this study seemed to get distressed upon learning the outcome of the diagnosis of the patient under their care. Some seemed to be at ease and few embraced the problem and decided to live with it. It is true that not all people react to the same event in the same manner. However, how one reacts to the outcome of PD diagnosis is likely to affect the subsequent actions she/he would take. Negative overreactions as well as unresponsiveness are likely to lead to negative aspects in the caregivers and compromise the cares they are supposed to provide to the patient [10]. Accepting the outcome of the diagnosis and remaining calm though less frequent may help individuals make right decisions as to what to do next. In accordance with this claim, Ferreira et al. [10], in their integrative review, have identified learning to act calmly, positive thinking, and more patience as key ingredients of positive aspects for caregivers of PD patients.

“Caring for a PD patient is a tough undertaking that demands patience.” This is another key theme that emerged from the present data. When asked to describe “what does it mean to be a PD patient caregiver” in one word or phrase, vast majority of the participants mentioned the word “difficult” or “tough” explicitly and implicitly. They described it as a tough responsibility that demands “patience.” This finding is consistent with findings of previous studies. For example, caring for a PD patient has been described as “burdensome”, “challenge,” and “overload” (see [10, 15] for the reviews) and “stressful” [2]. As this study highlighted, patience has been underscored as one of the positive aspects that people who care for PD patients need to possess (e.g., [10]).

This study also highlighted that caring for PD patients is a shared responsibility. When asked “are you the only caregiver of the PD patient?” vast majority of the caregivers said no pointing out that they do have at least one person who shares the responsibility of caring for the patient. This theme also persisted in another question, which asked the respondents to rate the supports they receive from different sources. Here, the participants rated family members and close relatives and friends as their first and foremost source of support echoing the finding that caring for PD patients is a shared responsibility. In fact, this is not surprising given the collective nature of Ethiopian societies. This finding is partly in line with that of Helen [7]. Having collected data from PD patients, caregivers, and neurologists, Helen [7] has come to conclude that while some patients did not have caregivers, others did have two or more family members and close others who used to care for the PD patients. Indeed, presence of multiple caregivers is desirable and is beneficial to both PD patients and their caregivers.

Another important theme that emerged from my data was that community's understanding of PD is very low and even distorted. In the participants' opinion, their community's understanding of PD is not only very low but also distorted. It is wrongly attributed to evil spirit, killing cat as a child, and witchcraft; it is also seen as a curse from God as a consequence of one's sins. In support of this finding, Helen [7] has found that PD was one of the diseases that are misunderstood in Ethiopia. Kibra [6] has rightly observed that society's association of PD with evil spirit has made PD patients self-ostracize instead of going out and seeking modern medication. Dotchin, Msuya, and Walker [16] maintained that cultural norms in Africa force PD patients and people with similar illnesses to isolate themselves and visit traditional healers before seeking modern medical help, which in turn leads to delay in diagnosis and intervention.

In connection to this, PD has neither specific name nor clear expression in local languages. In this regard, Kibra [6] has grieved the grave lack of written materials in languages spoken in Ethiopia and how this has made their efforts to inform very difficult. Actually, lack of clear expression for PD in local languages and written materials in any of the languages spoken in Ethiopia may reinforce lack of understanding and distortion of PD among the public and other stakeholders.

“Lack of sufficient support” from different sources is one of the challenges that the caregivers said to have faced. Particularly, they reported that they do not get adequate support from the larger community or from NGOs and government agencies that are supposed to provide key support to them. In this regard, Helen [7] has reiterated that PD is one of the noncommunicable diseases that has got no or little attention from governmental and nongovernmental organizations, donor agencies, and media outlets. Kibra [6] observes that, unlike other parts of the world, there are no charity organizations, foundations, or government agencies that work with PD patients in Ethiopia. Due to their limited movement, they are not only invisible but also voiceless. Overall, they are neglected and thus are not getting supports they deserved to get. Like this study, de Villiers et al. [2] found out that most supports to PD patients' caregivers come from family members. Dochtin et al. [16] noted that many chronic conditions including PD receive little recognition in developing world as a result to which services available to PD patients in Africa and their family are limited or nonexistent at all. In fact, support, be it social or professional support, is a protective factor against caregiving-related stress and lack of it can compromise the caregiving capacity of caregivers and the quality of care the patients receive from them.

Participants of this study pointed out that caregivers' compassion and patient's courageousness were what helped them to cope with the burden of caring for PD patients. It is true that compassionate caregivers are positive thinkers and positive thinking is one of the positive aspects of caring for patients with PD [10]. Compassionate caregivers are more likely to exercise more patience; they are more likely to be positive thinkers and resilient; they may have reasonably high self-esteem and self-efficacy. Furthermore, they are capable of generating more social support. These qualities (patience, positive thinking, high self-esteem and self-efficacy, and resilience) are attributes that being a good caregiver to patients with a PD requires. Research shows that such personal attributes are protective factors that shield caregivers against burdensome that originates from caring for people with chronic conditions including patients with PD (e.g., [17]). On the other hand, courageous patients with PD are resilient and are likely to elicit quality care from their caregivers and ease caregivers' burden. In fact, according to Ferreira et al. [10], the process of caring for patients with PD demands resilience on part of the caregivers and individuals with PD. Caregiving is a bidirectional process and therefore courageousness and resilience in the care receiver are unquestionably a key resource that protects the caregiver against caregiving-related burdensome and distress.

Finally, “shortage and expensiveness of PD drugs/prescriptions” was what has been lamented by the participants of this study. Kibra [6] has stated this problem as “…medicinal issue is what has been worrying me since my diagnosis with PD”(p.108). She has gone on explaining areas where medication for patients with PD is highly problematic: (1) lack of drugs/prescriptions in smaller doses; (2) lack of alternative drugs/prescriptions and enough number of specialist neurologists; and (3) expensiveness of the drugs available. For example, according to Kibra [6], the price of locally available PD drug hiked from 32 birr in 2014 to 117 in 2018 and patients with PD in Ethiopia are no more capable of affording for their medications. In fact, shortage of drugs and medical treatment is a common problem of developing world, though it is grave in sub-Saharan Africa as Dotchin et al. [16] noted. According to Dotchin et al., [16], only 12.5% of patients with PD in Africa have had access to drugs for PD compared to 79.1% in Europe. Overall, drugs for PD in Africa are not only unavailable but also unaffordable for those who are in a dire need of them.

## 5. Recommendations

Based on the key findings of this study, the following recommendations could be forwarded:

- Public/community awareness and knowledge of PD and patients with PD are very crucial. Therefore, concerted education and awareness campaigns and programmes need to be put in place to raise public/community awareness. This should include production and dissemination of reading materials in widely spoken languages in the country.
- What WHO [1] noted a decade ago is still a reality in poorer countries such as Ethiopia. In other words, there is still a grave lack of universal access to the presently available wide range of medications, surgery, and complementary therapies. Therefore, a lot needs to be done including training physicians and specialists and increasing the number of health facilities equipped to provide comprehensive health care to patients with PD and their families.
- Shortage and high cost of medication and prescription is what has been highly grieved by the participants of this study. Therefore, subsidized medication programmes for patients with chronic conditions in general and for patients with PD in particular should be set.
- Lack of adequate support to the family and caregivers of patients with PD from the community, governmental, and nongovernmental organizations and other stakeholders is what has been echoed in this study. Hence, concerted and integrated support is highly required to ease the burden and stress of caregivers and family members. Institutions and programmes that provide comprehensive care for the PD patients, their caregivers, and family members are highly desired. Psychoeducational programmes that empower patients with PD and their caregivers are crucial.
- Lastly, but importantly, research pertinent with PD, patients with PD and their caregivers, healthcare delivery to patients with PD, and so forth must be encouraged.

## 6. Conclusion

This research aimed at exploring, uncovering, and understanding experiences and perspectives of caregivers of patients with PD. To this end, it collected data from 20 caregivers who responded to a questionnaire that consisted of mostly open-ended questions that asked them to provide their own accounts of issues related to PD and caring for patients with PD. Analysis of the data yielded several themes including lack of sufficient support to the caregivers, caregivers' compassion and patient's courageousness, and shortage and expensiveness of PD drugs/prescriptions. To the researcher's best knowledge, this research is one of the first in its kind and has generated valuable information that has far-reaching implications to meet the unique needs of patients with PD and their caregivers and other family members in Ethiopia and beyond.

However, this research is not without limitations. The first clear limitation is that it generated data from only 20 participants. Second, it relied on self-reported data alone. Third, the data came from caregivers only. Albeit these and other potential limitations, this research has added a lot to our understanding of the experiences and perspectives of people who care for patients with PD and laid a good foundation for further research into issues related to patients with PD and their care. Future research would benefit from extensive and intensive data that would come from multiple sources of data through multiple techniques of data collection.

## Figures and Tables

**Table 1:** Demographic information of participants.

	Demographic characteristics				
Code	Age in years	Gender	Education	Civil status	Occupation
P01	14	Male	Primary	NA	Student
P02	25	Male	Primary	Single	Unemployed
P03	45	Female	Secondary	Married	Civil servant
P04	48	Male	College dip	Single	Self-employed
P05	50	Female	Secondary	Widow	Laborer
P06	36	Female	Primary	Married	Laborer
P07	36	Male	College dip	Single	Self-employed
P08	19	Female	Secondary	Single	Student
P09	20	Female	Uni. student	Single	Student
P10	60	Female	-* *-* *-	Married	Housewife
P11	34	Male	College dip	Single	Self-employed
P12	66	Female	-* *-* *-	Married	Housewife
P13	55	Female	Primary	Married	Housewife
P14	65	Female	Primary	Married	Housewife
P15	24	Male	Degree	Single	Self-employed
P16	21	Male	Secondary	Single	Civil servant
P17	40	Female	Primary	Single	Civil servant
P18	38	Female	Primary	Married	Unemployed
P19	19	Female	Uni. student	Single	Student
P20	22	Female	Degree	Single	Civil servant

NA, not applicable; P, participant; -* *-* *-, missing data.

## Data Availability

The data used to support the findings of this study are available from the corresponding author upon request.
